# Fine spatial-temporal density mapping with optimized approaches for many-core system

**DOI:** 10.3389/fnins.2025.1512926

**Published:** 2025-04-03

**Authors:** Song Wang, Yiyuan Gao, Bingfeng Seng, Jing Pei, Yuan Zhang, Jianqiang Huang

**Affiliations:** ^1^School of Computer Technology and Application, Qinghai University, Xining, China; ^2^Qinghai Provincial Laboratory for Intelligent Computing and Application, Qinghai University, Xining, China; ^3^Department of Precision Instrument, Center for Brain Inspired Computing Research (CBICR), Tsinghua University, Beijing, China; ^4^Qinghai Provincial Green Computing Power Engineering Technology Research Center, Xining, China

**Keywords:** many-core, spatial-temporal density mapping, memory management, spatial resource, computational speed

## Abstract

A fine mapping strategy is essential for optimizing the layout and execution speed of large-scale neural networks on many-core systems. However, the benefits of many-core systems diminish when applied to neural networks with significant data and computational demands, due to imbalanced resource utilization between space and time when relying on existing single spatial or temporal mapping strategies. To tackle this challenge, we introduce the concept of spatial-temporal density and propose a spatial-temporal density mapping method to fully leverage both spatial and computational resources. Within the framework of the proposed method, we further introduce two approaches: the Negative Sequence Memory Management (NSM) method, which enhances spatial resource (i.e. core memory) utilization, and the Many-core Parallel Synchronous (MPS) approach, which optimizes computational resource (i.e. core multiply and accumulate units, MACs) utilization. To demonstrate the superiority of these methods, the mapping techniques are implemented on our state-of-the-art many-core chip, TianjicX. The results indicate that the NSM method improves spatial utilization by a factor of 3.05 compared to the traditional Positive Sequence Memory Management (PSM) method. Furthermore, the MPS approach increases computational speed by 6.7% relative to the previously widely adopted pipelined method. Overall, the spatial-temporal density mapping method improves system performance by a factor of 1.85 compared to the commonly employed layer-wise mapping method, effectively balancing spatial and temporal resource utilization.

## 1 Introduction

**Figure 1:**
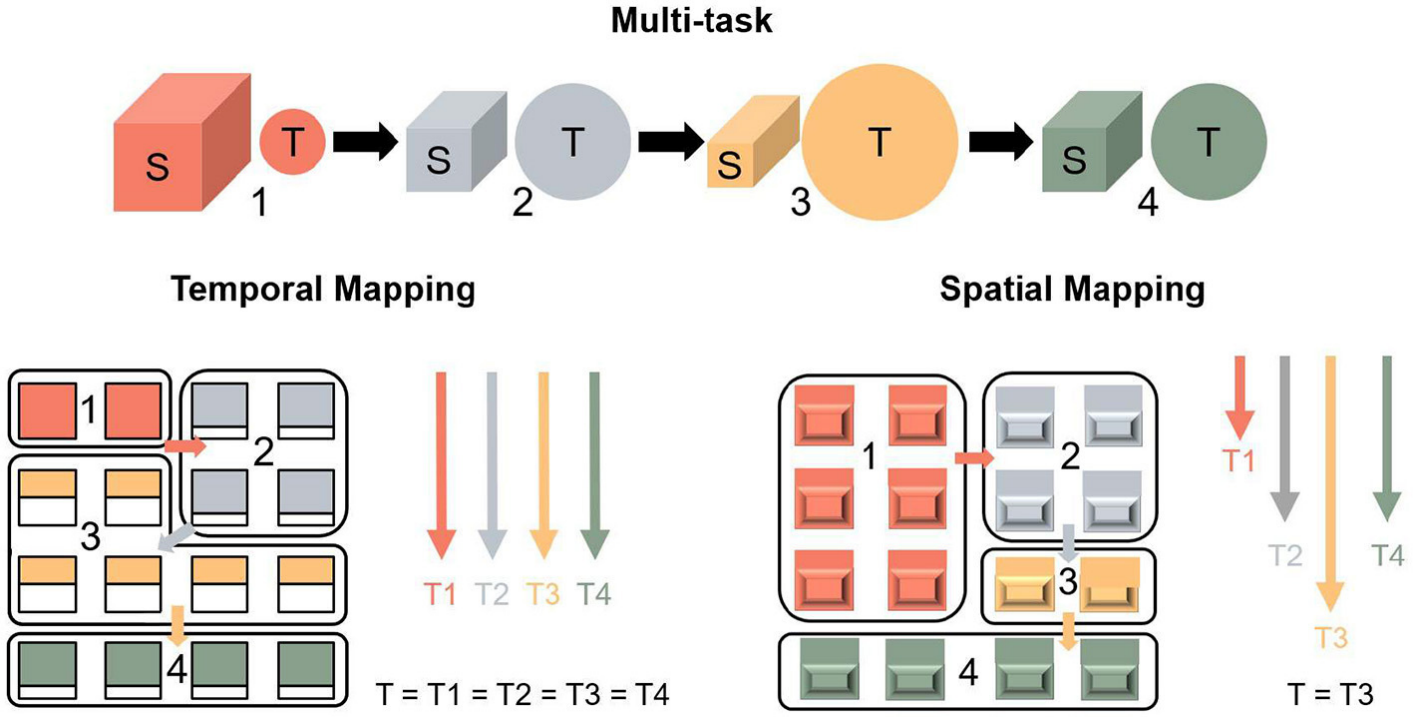
Illustration of the distinction between temporal mapping and spatial mapping scheme: the left of the figure shows that a large amount of data accesses the external memory in the temporal mapping, the right of the figure shows that the tail-latency exists in the spatial mapping which is determined by the longest time.

Recent many-core architectures have been widely adopted by accelerators (Shao et al., 2019; Chen et al., 2019; Modha et al., 2023) and neuromorphic chips (Sawada et al., 2016; Shen et al., 2016; Benjamin et al., 2014; Pei et al., 2019; Ma et al., 2022; Davies et al., 2018; Shrestha et al., 2024; Ambrogio et al., 2023; Le Gallo et al., 2023) due to their low power consumption and high parallelism. A crucial aspect of many-core systems involves mapping neural networks into pipeline groups, where each group is assigned a cluster of cores to handle computational tasks. In a many-core system, spatial resources correspond to the memory storage capacity of each core, which is closely associated with the number of parameters in a neural network. Computational resources refer to the number of multipliers and accumulators in each core, which are closely related to the computational workload of the neural network. In homogeneous many-core systems, the temporal and computational resources are consistent across cores. However, the distribution of parameters and computational workload between the layers of a neural network is imbalanced. Two common strategies to implement neural networks are temporal mapping and spatial mapping, as illustrated in Figure 1. In temporal mapping, tasks are executed through time slicing, with each Processing Element (PE) or core independently accessing data and taking on tasks according to their time complexity. However, this approach leads to data duplication between cores, resulting in inefficient utilization of spatial resources. Although this method eliminates tail latency between cores, it incurs significant data movement between cores and external storage due to the limited memory capacity of the cores, as shown on the left side of Figure 1. Alternatively, spatial mapping divides tasks according to spatial slicing, where clusters of cores are assigned tasks based on spatial volume (Ma et al., 2022). Although this method reduces data movement, it introduces tail latency across clusters, leading to inefficient utilization of computational (i.e., temporal) resources, as shown on the right side of Figure 1. Moreover, the layer-wise mapping approach, commonly employed in many-core systems (Zimmer et al., 2020; Chen et al., 2019; Pei et al., 2019; Le Gallo et al., 2023), integrates both temporal and spatial mapping strategies, offering improved mapping efficiency compared to single-method approaches. However, it still encounters issues of imbalance between spatial and computational resources, as inconsistencies in the partitioning scheme across layers may introduce latency during reshaping between adjacent layers. To tackle this challenge, we propose spatial-temporal density mapping to balance the utilization between the spatial resource and the computational resource.

Nevertheless, the spatial-temporal mapping scheme faces challenges like memory constraints and time delays. During the mapping process, spatial resources are prioritized when allocating cores. Because core memory space directly impacts data movement and memory access, which play a critical role in chip energy consumption and memory footprints (Han et al., 2016; Chen et al., 2016). In neural networks, most historical and intermediate data must either be discarded or updated during computation (Hu et al., 2021), allowing memory space to be reused once it is freed. Current research efforts have largely focused on reducing memory footprints and data movement in traditional hardware systems. Techniques such as fine-grained memory management (Nie et al., 2022), reinforcement-based memory management for GPUs (Liu et al., 2021), and machine intelligence-driven hybrid memory management (Doudali and Gavrilovska, 2022) have shown promising results. However, there remains a notable gap in research addressing storage management strategies tailored specifically for many-core systems. When mapping large neural networks onto hardware, partitioning is necessary due to the limited memory and MACs available on individual cores. In practice, the input channel (*C*_*in*_) of the neural network is typically selected for partitioning, as the channel dimension is strongly correlated with the computational load, including multiplications and accumulations (Xie et al., 2017). Partitioning along the *C*_*in*_ dimension results in the generation of partial sums (Psums) across multiple cores. To ensure computational precision, hardware architectures such as TianjicX (Pei et al., 2019) and Simba (Shao et al., 2019) expand the bit-width of these Psums. However, the increased width of Psums necessitates their accumulation across cores or PEs, leading to significant communication latency. Given these two aspects, it is critical to develop optimized methods for memory management and Psums computation to improve the utilization of both spatial and computational resources, thus enhancing memory efficiency and reducing computational latency.

In this work, we propose a fine-grained spatial-temporal density mapping scheme to balance the utilization of spatial and computational resources. Decentralized many-core systems (Lin et al., 2018; Shen et al., 2016; Benjamin et al., 2014; Pei et al., 2019; Ma et al., 2022; Zhong et al., 2024) are well-suited for executing multiple neural networks concurrently, enabling the simultaneous exploitation of both spatial and temporal complexities. To demonstrate the feasibility of our proposed mapping scheme, we leverage our state-of-the-art many-core chip, TianjicX (Ma et al., 2022). TianjicX chip is capable of spatial-temporal elasticity, effectively executing and coordinating multiple tasks in parallel. Various neural network models have been successfully deployed on TianjicX chip (Zheng et al., 2024; Wu et al., 2024), which has also been utilized for applications such as gaming and place recognition in edge robotics (Ma et al., 2022; Yu et al., 2023). To further explore the advantages of spatial-temporal density mapping, we introduce Negative Sequence Memory Management (NSM) method to enhance spatial resource utilization, and Many-core Parallel Synchronous (MPS) approach to optimize computational resource utilization. We conduct a theoretical analysis of the proposed spatial-temporal density mapping scheme and applied it to map the typical neural network architecture, ResNet-50, onto TianjicX hardware. Experimental results show that our method outperforms traditional mapping approaches, demonstrating its superiority in terms of efficiency.

The remainder of this article is organized as follows: Section 2 provides background on spatial and temporal mapping, along with memory space management and partial sum computation. Section 3 details the proposed approach, including the theoretical analysis of spatial-temporal density mapping, NSM, and MPS. The hardware implementation of TianjicX is discussed in Section 4. Section 5 presents the experimental results of the proposed mapping method, followed by a comparative analysis with traditional mapping approaches. Finally, Section 6 concludes the paper and offers insights for future work.

## 2 Related works

### 2.1 Spatial and temporal mapping

The hardware architecture dictates the mapping scheme. Neuromorphic chip architectures based on crossbar arrays employ mapping schemes to enhance memory space utilization (Amir et al., 2013; Cui et al., 2022; Wei et al., 2022; Rueckauer et al., 2022; Zou et al., 2021). For instance, a compiler has been proposed that utilizes a greedy layer-wise optimization algorithm and connection sharing to minimize the duplication of weight kernels in convolutional topologies for the Loihi core (Davies et al., 2018; Rueckauer et al., 2022). This approach achieved near-optimal space resource utilization of 80 % across 16 chips for a 28-layer network. Similarly, FangTianSim has been introduced, which flattens input images and output neurons into one-dimensional arrays for mapping spiking neural networks (Wei et al., 2022). This method aims to improve the utilization of resistive random-access memory (RRAM) in crossbar structures. Additionally, channel-major search and square-major search algorithms have been proposed to ensure high resource efficiency and compactness in hardware modules (Zou et al., 2021). These algorithms also introduced density metrics for axons, neurons, and synapses as practical evaluation criteria for assessing crossbar resource efficiency. In addition to spatial mapping, some neuromorphic chips employ temporal mapping schemes. A loop representation with simulated annealing has been used to place local structures on hardware, minimizing communication hops and optimizing energy costs (Cui et al., 2022). Another approach involves a fully-unfolded temporal mapping that reuses limited crossbar resources through time-division multiplexing (Esser et al., 2016). However, this method incurs substantial memory overhead despite achieving high computational parallelism. To balance spatial and temporal resource utilization, a semi-folded mapping paradigm has been proposed that strategically allocates resources to optimize overall efficiency (Deng et al., 2018). In a recent study, a neuromorphic chip with 1024 scalable cores was designed, and mapping experiments demonstrated that the semi-folded mapping strategy significantly reduced core overhead by a factor of 0.07, while only moderately decreasing inference throughput by a factor of 0.04 (Zhong et al., 2024). Despite these advancements, the challenge of balancing spatial and temporal resource utilization in crossbar-based neuromorphic architectures remains a critical issue. While substantial research has focused on improving spatial resource utilization through various mapping schemes, the exploration of temporal efficiency in artificial neural networks (ANNs) is still relatively limited. Similarly, mapping ANNs onto many-core architectures also faces the challenge of unbalanced utilization efficiency between spatial resources and computational resources. However, research on effectively balancing spatial-temporal resource utilization for ANNs remains underexplored to date.

**Figure 2:**
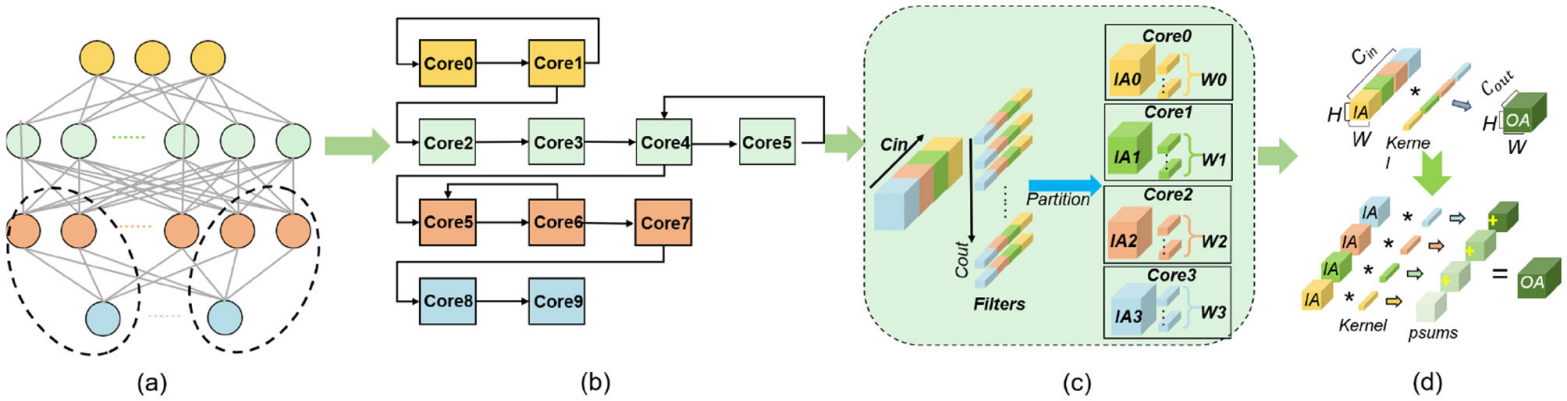
The process of generating partial sum: **(a)** neural network with multi-layers; **(b)** layer-wise mapping by temporal mapping or spatial mapping; **(c)** partitioning the neural networks; **(d)** process of resulting Psums.

On the other hand, most many-core designed without crossbar achitecture adopts layer-wise approach for artificial neural network mapping (Chen et al., 2019; Pei et al., 2019; Le Gallo et al., 2023; Zimmer et al., 2020), as illustrated in Figures 2a, b in a pipelined manner. The distribution of parameters and computational workload across each layer is imbalanced, leading to tail latency being determined by the longest execution time among the allocated cluster cores. While TianjicX architecture (Ma et al., 2022) represents a pioneering effort in integrating spatial-temporal mapping for multi-task processing, its underlying spatial-temporal coordination mechanism has not been thoroughly explored, particularly in terms of strategies for mitigating tail latency. To address the critical challenge of optimizing spatial-temporal resource utilization in ANNs, this paper proposes a novel spatial-temporal density mapping framework.

### 2.2 Management of the memory space

Efficient memory management systems are crucial for minimizing memory footprint and reducing data movement. Existing memory management systems are predominantly designed for the Von Neumann architecture, which relies on external memory. The vDNN architecture (Rhu et al., 2016) introduces a swap strategy and employs a layer-wise memory management approach. Several studies (Huang et al., 2020; Jiang et al., 2019; Wahib et al., 2020) address GPU memory footprint reduction using coarse-grained methods. These methods typically involve swapping or recomputing data during the backward phase and evicting tensors during the forward phase. Many of these approaches (Huang et al., 2020; Chen et al., 2018; Xiao et al., 2020) focus on tensor-wise memory management. However, this approach limits the flexibility of the swapping policy (Nie et al., 2022). To overcome this limitation, a fine-grained memory management system based on tensor splitting has been proposed, aiming to alleviate memory bottlenecks while maintaining neural network training efficiency (Nie et al., 2022).

Additionally, various other memory management techniques have been developed, such as reinforcement-based methods for class-incremental learning, holistic approaches for GPU systems, and layer-conscious memory management frameworks for FPGA-based accelerators. Despite these advancements, many-core systems, unlike the Von Neumann architecture, typically lack external memory. As a result, methods for memory management in decentralized many-core systems remain underexplored.

**Figure 3:**
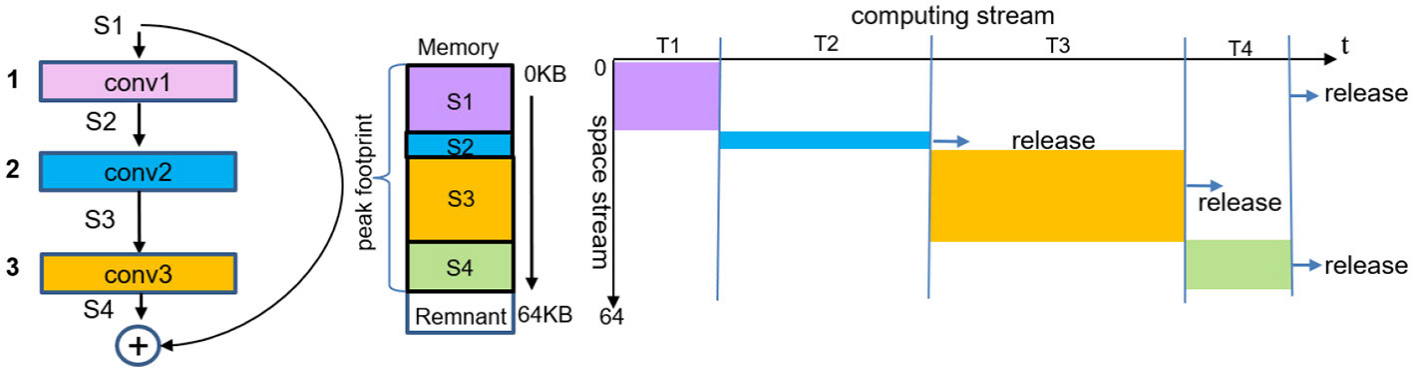
Positive sequence memory management for multi-layers. The data and the start address of each layer are stored along the direction of address increase. The *S*_1_ has a forward propagation due to the residual connection. And the space of the *S*_1_ can be released after the phase of the *T*_4_. The *S*_2_ can not be reused in the phase *T*_3_ in real time for the space of the *S*_3_ is larger than that of the *S*_2_.

TianjicX chip, a many-core system, utilizes Positive Sequence Memory Management (PSM), as shown in Figure 3. However, this approach struggles with efficient memory space reuse. For instance, the space of *S*_3_ is larger than the combined space of *S*_1_ and *S*_2_, so the *S*_2_ space can only be reused when both *S*_1_ and *S*_2_ spaces are released. To address these limitations and improve memory utilization in many-core systems, we propose a Negative Sequence Memory Management (NSM) approach.

### 2.3 Partial sum computing

As shown in Figures 2c, d, partial sums (Psums) are prevalent in the AI hardware computation process, particularly when the *C*_*in*_ dimension is selected for partitioning to address limited memory capacity (Shao et al., 2019; Wang et al., 2021; Wu et al., 2020). To reduce both the number of parameters and computations, some researchers have adopted grouped convolution (Xie et al., 2017; Howard et al., 2017; Zhang et al., 2019; Wang et al., 2019), where Psums are directly activated on each core of the GPU platform without aggregation. However, this approach entails a quantifiable trade-off in accuracy (Howard et al., 2017). Empirical validation reveals a 1% reduction in ImageNet classification accuracy when employing direct activation of depth-wise separable convolutions, as opposed to full convolutions where partial sums (Psums) are activated post-aggregation.

To maintain accuracy, most accelerators perform activation after Psums aggregation and expand the bit-width of Psums. To address the challenges posed by large-bit-width Psums, several accelerators adopt a Pipelined Manner (PM) (Shao et al., 2019; Chen et al., 2019; Jouppi et al., 2017; Sze et al., 2017; Yin et al., 2017; Kung et al., 2019). In this approach, Psums are propagated through the processing element (PE) array or cores (Deng et al., 2020) during convolution or Matrix-Vector Multiplication (MVM) operations, which enhances data reuse and reduces the need for memory bandwidth.

TianjicX neuromorphic chip (Pei et al., 2019; Wang et al., 2021) adopts cluster cores with a dedicated function for managing Psums through Vector-Vector Accumulation (VVA). However, this configuration introduces latency between the VVA cores and other cores within TianjicX chip. To mitigate the latency associated with computing Psums, we propose the Many-core Parallel Synchronous (MPS) approach for Psums computation.

## 3 Motivation and approach

### 3.1 Spatial-temporal density mapping

TianjicX chip can support flexible mapping schemes, such as temporal mapping, spatial mapping, and spatial-temporal mapping. Firstly, we define a concept of spatial-temporal density (ρ) for a computing core. The spatial-temporal density can be described as the Calculation Amounts (CA) per unit space (S) of a core. The spatial-temporal density of a core can be described by Equation 1:


(1) $$\begin{array}{l} \rho_{i}=\frac{CA_{i}}{S} \end{array}$$

where the *i* represents the *i*-th core. Assuming *N* tasks are assigned to *M* cores, it can be described by Equation 2:


(2) $$\begin{array}{l} \overset{M}{\underset{i=0}{\sum }}{CA}_{i}=\overset{N}{\underset{j=0}{\sum }}{OPs}_{j} \end{array}$$

where the OPs represent the operations of the task. Therefore, the variance of the core density σ_ρ_ can be described as follows Equation 3:


(3) $$\begin{array}{l} \sigma_{\rho }=\frac{\overset{M}{\underset{j=0}{\sum }}{\left(\rho_{j}-\bar{\rho }\right)}^{2}}{M} \end{array}$$

**Figure 4:**
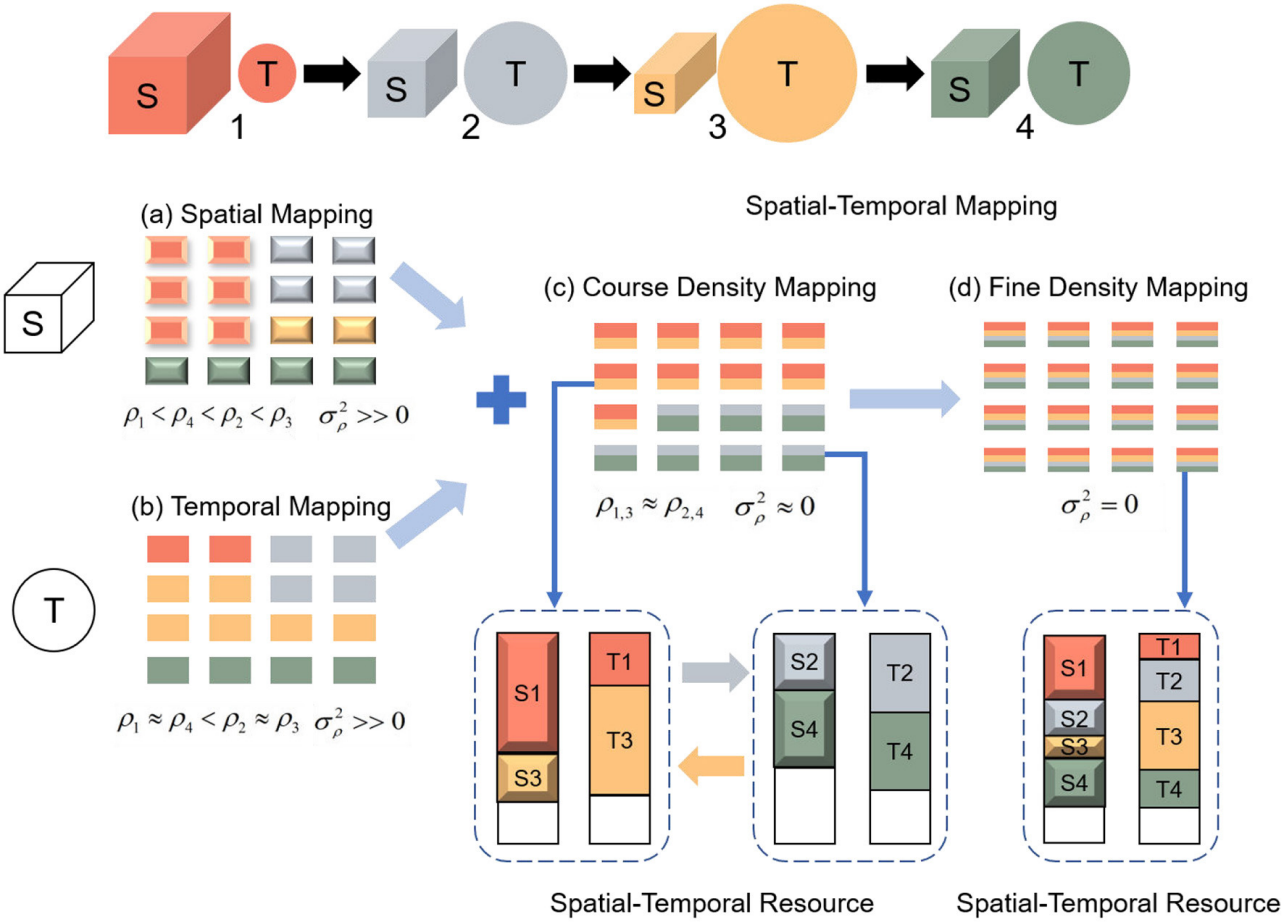
TianjicX supports flexible mapping. **(a)** Spatial mapping allocates cores according to the parameters of the task; **(b)** temporal mapping allocates cores according to the operations of the task; **(c)** coarse spatial-temporal density mapping allocates cores according to both the parameters and operations naively; **(d)** fine spatial-temporal density mapping.

As shown in Figure 4a, the density of each cluster core varies under spatial mapping. Figure 4b illustrates that the mapping scheme may fail if the first task occupies the largest memory space, requiring the allocation of 9 cores. While temporal mapping reduces latency, it leads to inefficient memory utilization. In contrast, spatial-temporal mapping effectively leverages the core density. The Tianjicat (Ma et al., 2022) adopts a coarse density mapping scheme, as depicted in Figure 4c.

To further optimize spatial-temporal density, we propose a fine spatial-temporal density mapping strategy. As shown in Figure 4d, this approach evenly distributes the operations and parameters of each layer across 16 cores. Notably, in this configuration, the density ρ of each core is uniform, and the standard deviation σ_ρ_ is zero, as observed in Figure 4d.

In the fine spatial-temporal density mapping scheme, the output activation for each layer does not require communication, since the partitioning of each layer is consistent and the output activation is stored locally. Consequently, reshaping latency caused by partition mismatches and communication latency between adjacent layers are eliminated. Moreover, by reusing the space of the preceding layer's input activation, memory consumption is further reduced.

### 3.2 Memory management for many-core system

**Figure 5:**
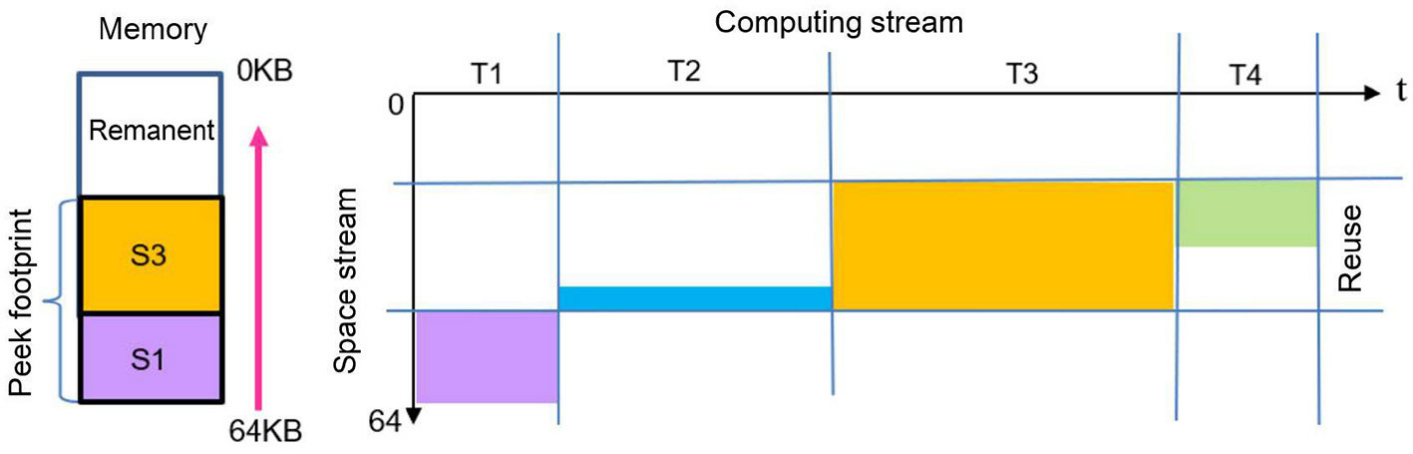
Negative sequence memory management for multi-layers. The data is stored along the direction of address increasing. While the start address of each layer is stored along the direction of the address decreasing. The space of the post layer can be reused as that of the current layer in real-time.

In deep neural networks, the input to a given layer is the output of the preceding layer, and pipelined execution occurs between adjacent layers. Since activation data must be continuously updated, the memory allocated for output activations can be dynamically reused for input activations in real time, provided there is no data scrambling. To enhance memory reuse efficiency, we propose a negative sequence memory management (NSM) strategy for multi-layer execution, as illustrated in Figure 5.

As shown in Figure 5, the memory allocated to *S*_1_ is not immediately reused during forward propagation. However, the memory regions allocated to *S*_2_ and *S*_4_ can be efficiently reused in real time through the NSM mechanism. Once *S*_1_ completes its forward propagation and is released, its memory can also be reused. Compared to conventional positive sequence memory management, NSM effectively reduces the peak memory footprint, which is determined by the combined memory usage of *S*_1_ and *S*_3_, leading to improved memory efficiency.

In order to avoid a scrambling between the input activation and output activation in real-time, the relationship of start address between two adjacent layers can be described as follows:

if (*V*_*i*+1_ > *V*_*i*_)


(4) $$\begin{array}{l} Addr_{i+1}=Addr_{i}-\left(V_{i+1}-V_{i}\right)-const \end{array}$$

else


(5) $$\begin{array}{l} Addr_{i+1}=Addr_{i}-const \end{array}$$

where the *Addr*_*i*_ represents the address of input activation or intermediate data in the *i*-th layer, the *V*_*i*+1_ represents the volume of the space parameters, the *const* is an address constant which is determined by the hardware. The scrambling of space between the adjacent layers can be eliminated by adjusting the value of *const*.

### 3.3 Many-core Parallel Synchronous (MPS) computing partial sum

**Figure 6:**
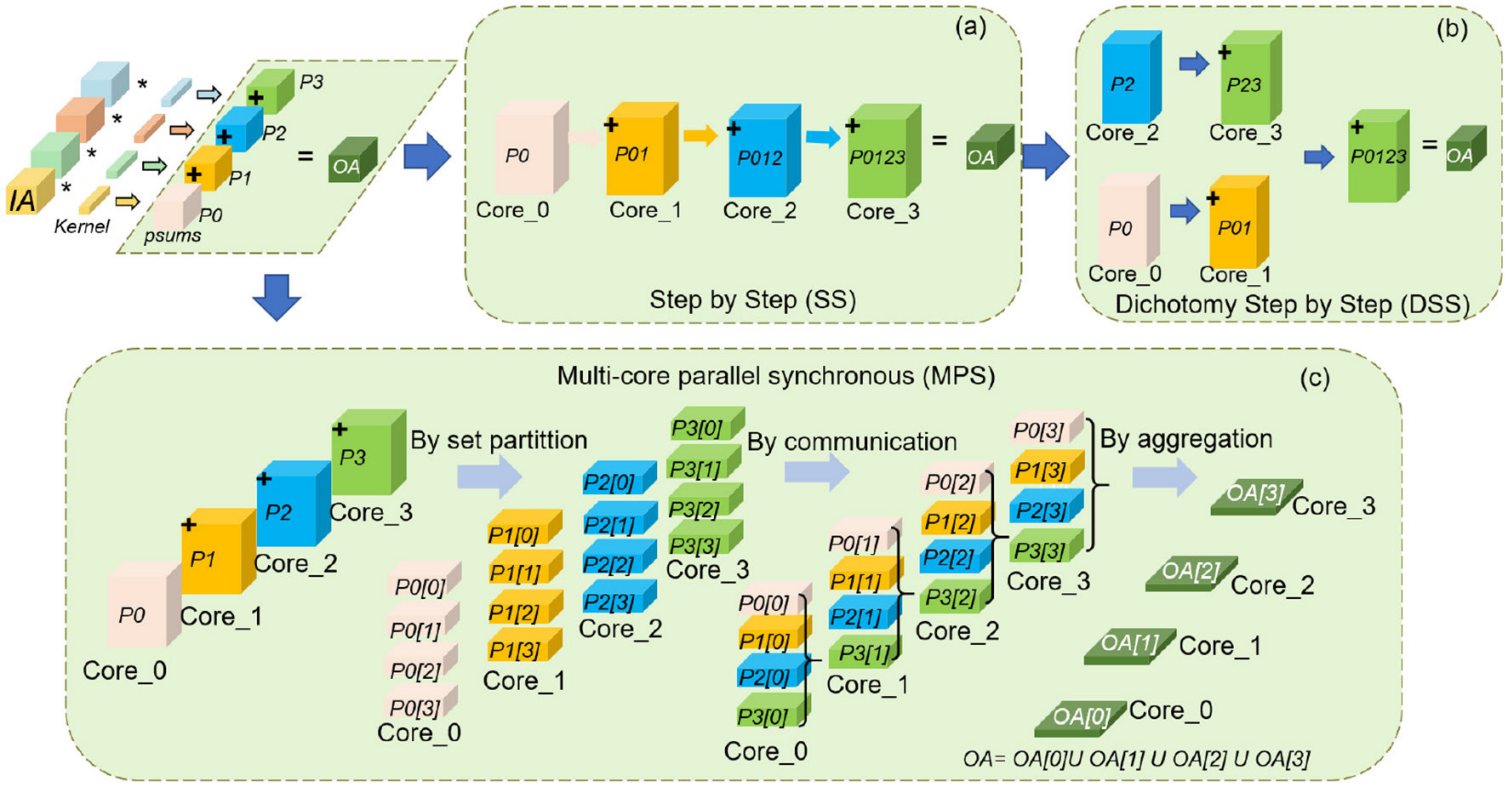
Illustration of supporting flexible methods for computing Psums: **(a)** Step-by-Step (SS) method; **(b)** Dichotomy Step-by-Step (DSS) method proposed by wallace tree; **(c)** Many-core Parallel Synchronous (MPS) method.

After allocating spatial-temporal resources to each cluster core, TianjicX system proceeds with neural network computation. As previously discussed, Psums must be efficiently managed during this process. TianjicX supports multiple approaches for addressing Psums, including the Step-by-Step (SS) method, the Dichotomy Step-by-Step (DSS) method, and the Many-core Parallel Synchronous (MPS) method. When using a *C*_*in*_ partitioning scheme with *M* = 4 groups, these methods are illustrated in Figure 6. Notably, the SS and DSS methods operate asynchronously, leading to inefficient utilization of computational resources, as some cores remain idle during Psums processing.

To achieve higher computational efficiency and minimize resource wastage, we adopt the MPS method. The detailed process is illustrated in Figure 6c. In this approach, each core partitions its assigned Psums into *M* groups, corresponding to the number of *C*_*in*_ partitioning groups. This process can be formally expressed as Equation 6.


(6) $$\begin{array}{l} \forall i\neq j,\;\cup_{i=1}^{M}P_{n}\left[i\right]=P_{n},\;P_{n}\left[i\right]\cap P_{n}\left[j\right]=\emptyset \end{array}$$

where *n* represents the *n*-th core. Secondly, the partitioning Psums sets of each core are communicated to other cores concurrently based on their corresponding serial numbers. Finally, each core aggregates Psums synchronously after communication. The aggregation and results of each core can be described as Equations 7, 8.


(7) $$\begin{array}{l} \forall n,OA\left[n\right]=\overset{M}{\underset{j=0}{\sum }}P_{j}\left[n\right] \end{array}$$

(8) $$\begin{array}{l} \forall i\neq j,n,\;\cup_{n=1}^{M}OA\left[n\right]=OA,\;OA\left[i\right]\cap OA\left[j\right]=\emptyset \end{array}$$

where the *OA*[*n*] represents the *n*-th set of the output activation. By the method of MPS, each core can compute 1/*M* parts of output activation set concurrently. Throughout the entire addressing Psums process, all the cores work in communication and computation synchronously all the time. Therefore, the computation speed of MPS is faster than that of SS or DSS.

## 4 Implementation details

**Figure 7:**
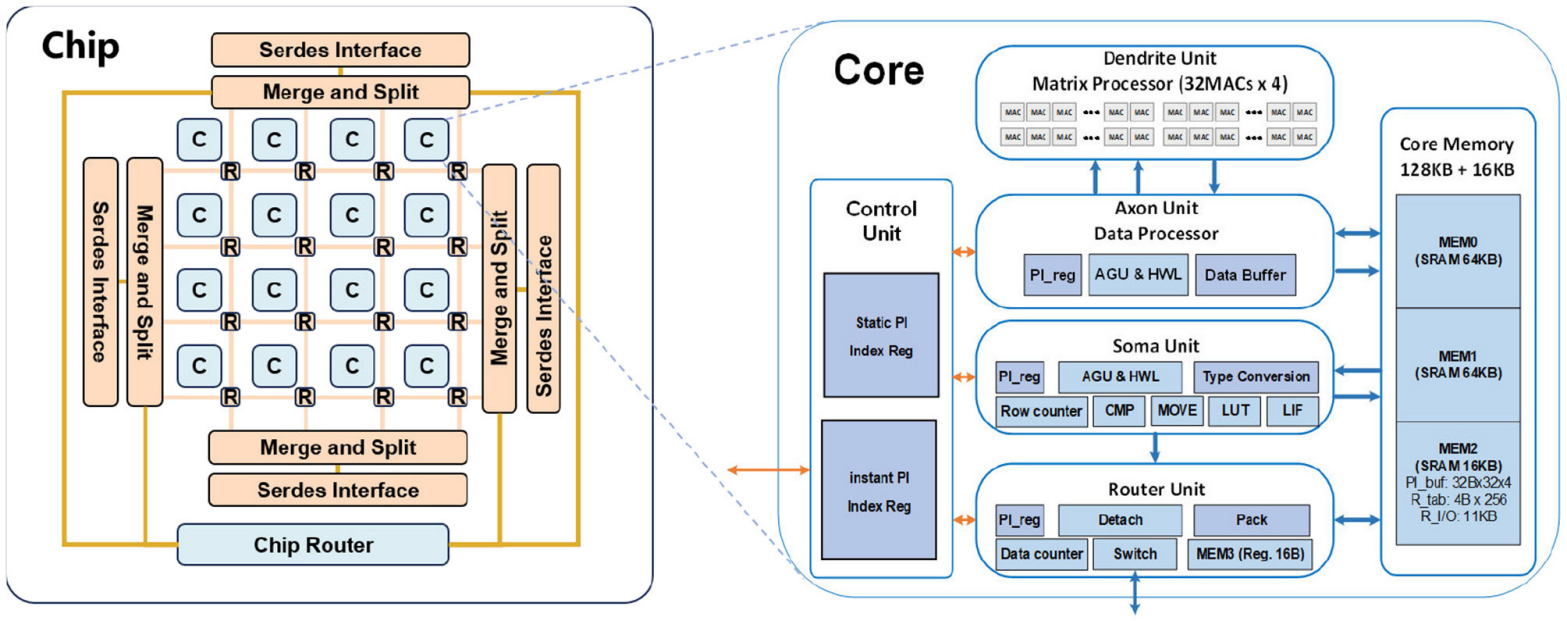
The architecture of the TianjicX based on a many-core design implemented with digital circuits.

TianjicX chip was fabricated using the UMC 28-nm High Performance Compact Plus (HPC+) CMOS process and assembled in an FPGA-225 package. Prior studies have demonstrated its outstanding performance in terms of fundamental characteristics, computational capabilities, and power efficiency (Ma et al., 2022). TianjicX employs a decentralized many-core architecture comprising 160 functional cores, as shown in Figure 7. Additionally, the chip supports flexible mapping strategies and partitioning methods, allowing for optimized neural network execution. TianjicX architecture employs a fully digital design featuring a non-crossbar memory through innovative memory addressing schemes. Each computational core functions as a reconfigurable processing engine capable of executing vector-matrix multiplication (VMM), vector-vector multiplication (VVM), and vector-vector accumulation (VVA) operations through synergistic collaboration of its six functional modules: controller, axon, dendrite, dual-port memory (2 × 64KB), soma, and router. The axon module specializes in the orchestration of tensor data and input buffering for dendrite operations, while preprocessed input vectors and synaptic weights are managed by an external controller and stored in the dual memory banks of the core. The dendrite module incorporates a high-throughput arithmetic unit with 128 parallel 8-bit multipliers coupled with 128 32-bit signed accumulators, enabling simultaneous multiply and accumulate (MAC) operations. Post-processing operations including nonlinear activation functions (ReLU), leakage integration mechanisms, and spiking neural models (LIF) are implemented in the soma module through configurable data transformation pipelines. The axon and soma operations employed in the subsequent experiments are listed in Table 1. The router is responsible for data communication between each core. The architecture implements unified memory addressing with dynamic resource allocation, allowing flexible memory partitioning and shared access across computational modules. This memory virtualization scheme supports various neural network paradigms through software-defined memory mapping, enabling efficient execution of neural networks.

**Table 1:** Axon and soma operations.

**# Uint**	**Operation**	**Defination**
Axon	VMM	y = w · x
Axon	VVA	y = $\underset{i}{\sum }x_{i}$
Soma	Relu	y = max(x,0)

**Figure 8:**
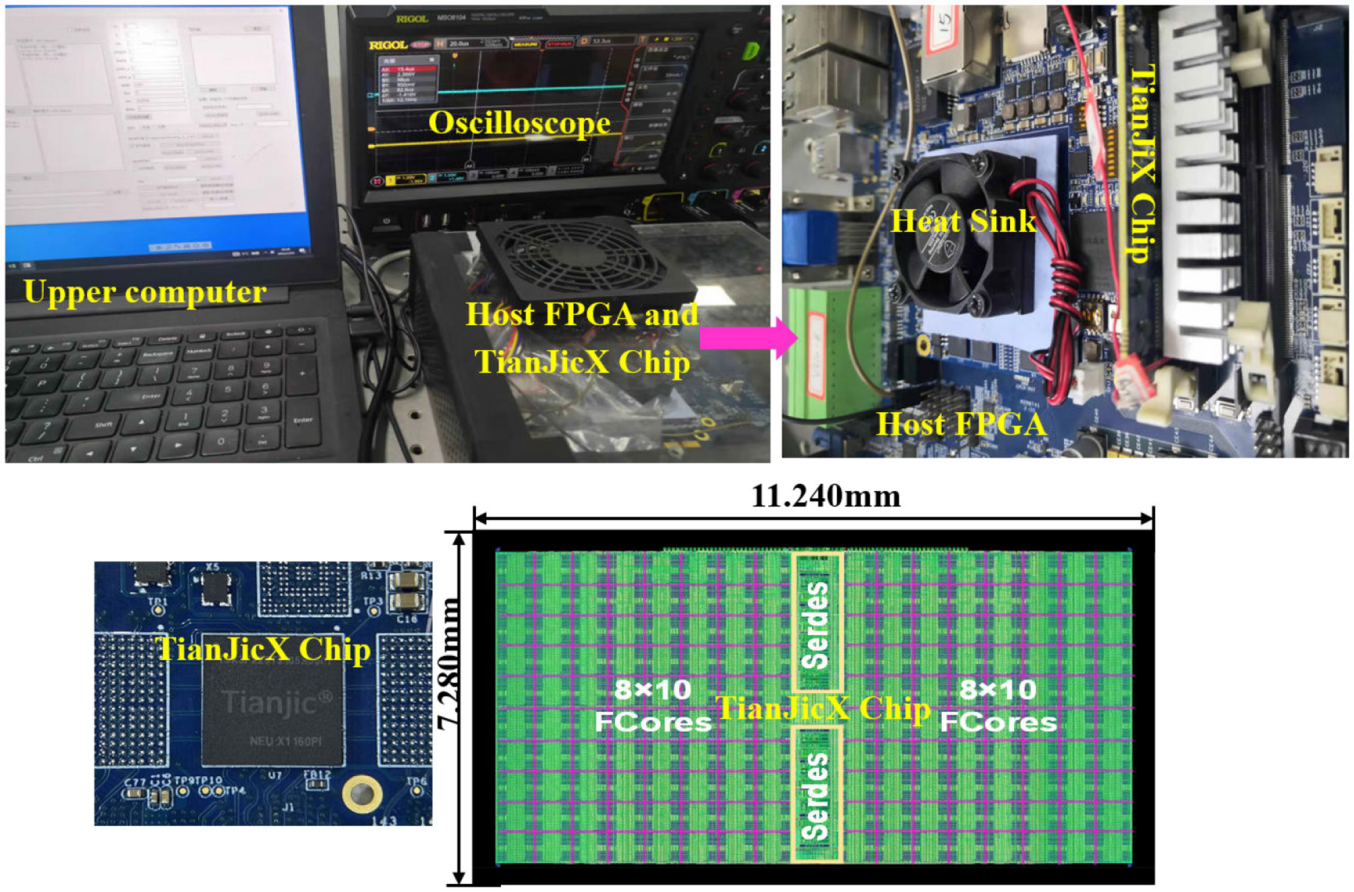
Testing system based on TianjicX neuromorphic chip.

The experimental setup includes an Intel Arria 10 FPGA, a host computer, TianjicX chip, and an oscilloscope, as depicted in Figure 8. Neural network parameters and inputs are configured and downloaded onto the chip via dedicated software on the host computer. Execution time is measured using a RIGOL MSO8104 oscilloscope. Prior to deployment on hardware, experiments are first simulated using TianjicX simulator, which faithfully replicates the real chip's behavior. This simulation system employs a dual-verification mechanism: the Behavioral Simulator and the Cycle-Accurate Simulator, which are implemented in different programming languages. The simulation results are deemed reliable only when the outputs from both levels of simulators are completely consistent. Once validated in simulation, the corresponding configuration files are downloaded to the chip for final execution.

**Figure 9:**
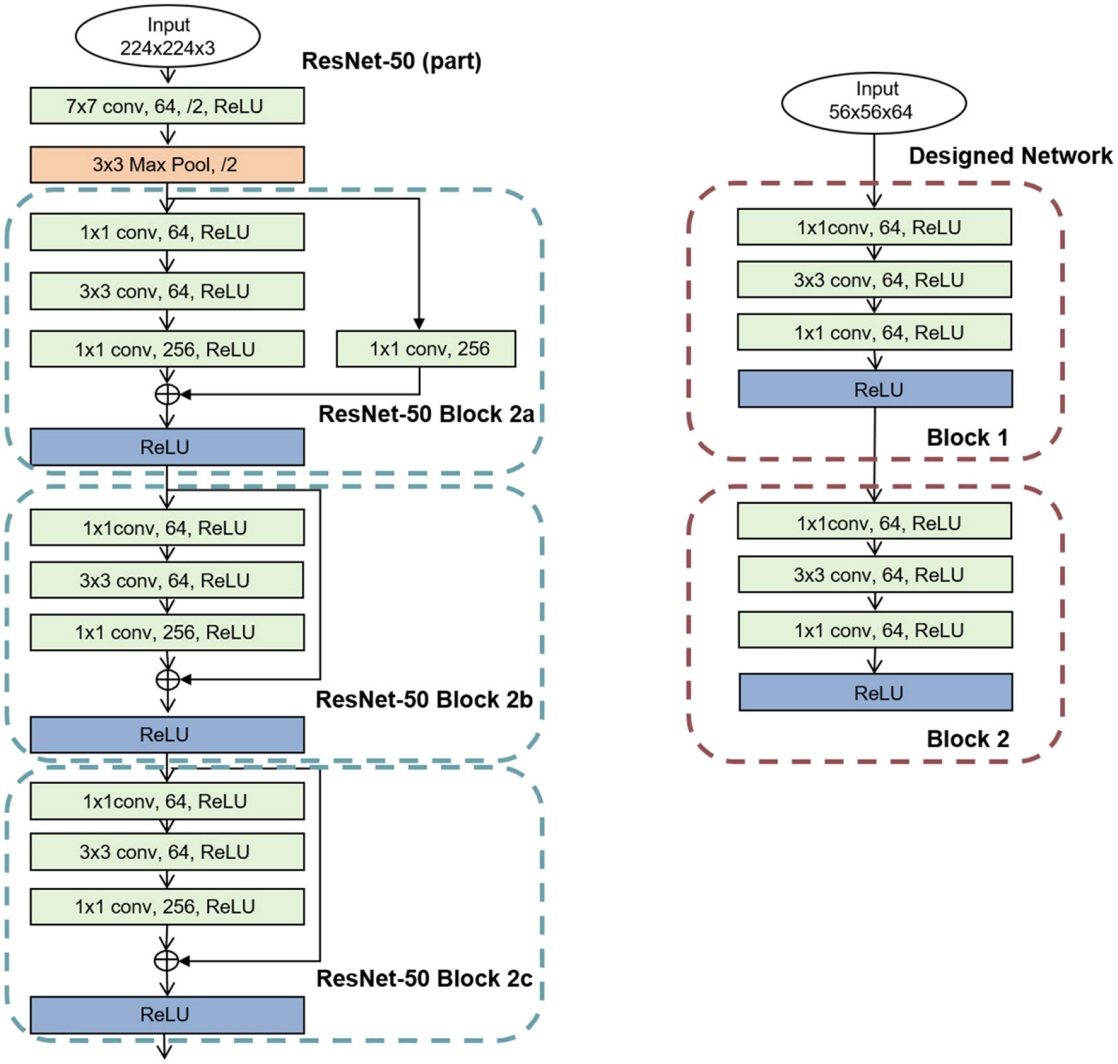
A part blocks of ResNet-50 network and a custom-designed network.

The ResNet-50 is often adopted to benchmark by many hardwares (Myung et al., 2021; Zimmer et al., 2020; Jouppi et al., 2021), which contains basic operator of Conv, Pooling, Skip and Relu. First, to evaluate the effectiveness of the proposed negative sequence memory management, all blocks of ResNet-50 are mapped onto the cores of TianjicX. Second, to assess the performance improvements achieved by the Many-core Parallel Synchronous (MPS) method, we conduct simulations comparing SS, DSS, PM, and MPS approaches. Finally, to demonstrate the benefits of fine spatial-temporal density mapping, we implement two neural network configurations on TianjicX chip: (1) a network consisting of two blocks (2b, 2c) from ResNet-50 and (2) a custom-designed network derived from the first configuration by removing residual connections. The parameters of the designed network are summarized in Figure 9.

## 5 Experiments and analysis

### 5.1 Simulation of the memory management

**Figure 10:**
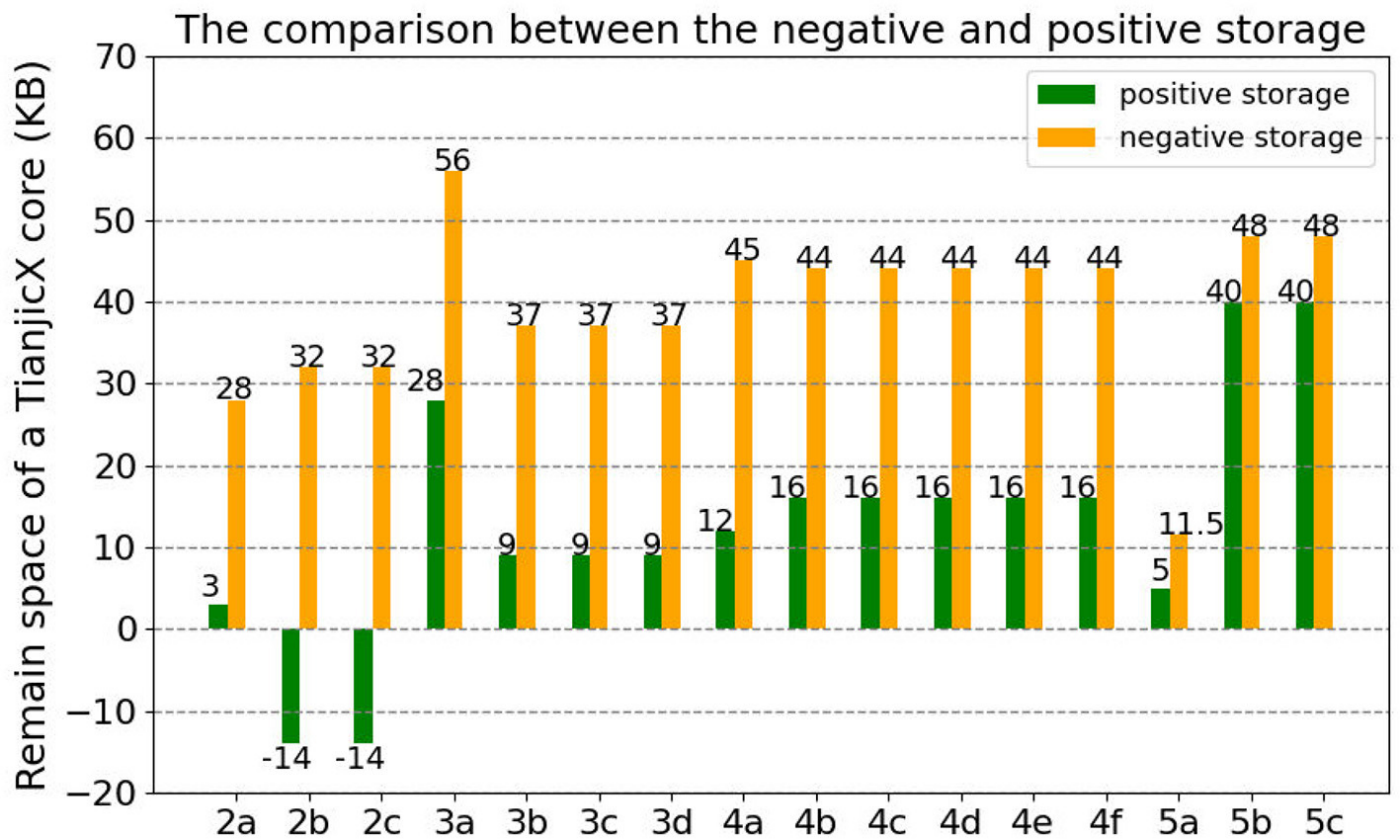
The space utilization of a core by the negative sequence memory management and the positive sequence memory management.

To evaluate the space utilization of the negative sequence memory management mechanism, all blocks of ResNet-50 are partitioned and mapped onto TianjicX cores. Both negative sequence and positive sequence memory management approaches are tested separately. The analysis results are presented in Figure 10.

As illustrated in Figure 10, our proposed NSM mechanism exhibits superior memory efficiency when compared to the conventional PSM approach. The experimental results yield three key observations: Firstly, the implementation of PSM for Blocks 2b and 2c leads to a negative residual memory capacity of -14 KB, signifying memory overflow and the necessity for additional core allocation to fully map the network. Secondly, NSM achieves its maximum optimization in Block 4a, where the residual memory capacity reaches 45 KB, marking a 3.75-fold improvement over the 12 KB achieved by PSM. Thirdly, a system-level analysis across all 17 benchmark blocks demonstrates that NSM increases the total available memory from 204 KB with PSM to 631.5 KB, which represents an average enhancement of 3.05 times.

Since NSM enables real-time memory release, it enhances memory utilization compared to PSM. By adopting NSM, input activation and output activation can be updated dynamically, allowing memory to be predominantly allocated for weight storage. Consequently, the weights of multiple tasks can be accommodated within the memory of a single core, further optimizing resource efficiency.

### 5.2 Simulation and analysis for the MPS

To evaluate the performance of MPS in computing partial sums (Psums), we conducted a simulation to compare the computational efficiency of various methods. Based on the computation process of Psums, all methods can be divided into two phases: the communication phase and the computation phase. Let *t*_1_ and *t*_2_ denote the execution times of the communication phase and computation phase, respectively.

We assume that the number of cores is *m*, which corresponds to the number of groups in the partitioning of *C*_*in*_. The amount of data handled by each core during communication is denoted as *X* (in KB). The hardware processing speed for data communication and data addition is represented by *K*_1_ (in B/s) and *K*_2_, respectively.

By employing 8-bit integers for weights and activations, and 32-bit integers for Psums, the total execution time for the SS, DSS, PM, and MPS methods can be computed as follows:


(9) $$\begin{array}{l} \begin{array}{c} t_{SS}=t_{1}+t_{2}\\ =\left(m-1\right)\frac{X}{K_{1}}+\left(m-1\right)\frac{X}{K_{2}}\\ =\left(m-1\right)X\frac{K_{1}+K_{2}}{K_{1}K_{2}} \end{array} \end{array}$$

(10) $$\begin{array}{l} \begin{array}{c} t_{DSS}=t_{1}+t_{2}\\ =\left[\log_{2}\left(m-1\right)+1\right]\frac{X}{K_{1}}+\left[\log_{2}\left(m-1\right)+1\right]\frac{X}{K_{2}}\\ =\left[\log_{2}\left(m-1\right)+1\right]X\frac{K_{1}+K_{2}}{K_{1}K_{2}} \end{array} \end{array}$$

(11) $$\begin{array}{l} \begin{array}{c} t_{PM}=t_{1}+t_{2}\\ =\frac{X}{K_{1}}+\frac{X}{K_{2}}\\ =X\frac{K_{1}+K_{2}}{K_{1}K_{2}} \end{array} \end{array}$$

(12) $$\begin{array}{l} \begin{array}{c} t_{MPS}=t_{1}+t_{2}\\ =\left(m-1\right)\frac{X}{K_{1}m}+\left(m-1\right)\frac{X}{K_{2}m}\\ =\left(m-1\right)X\frac{K_{1}+K_{2}}{mK_{1}K_{2}} \end{array} \end{array}$$

(13) $$\begin{array}{l} \frac{t_{PM}-t_{MPS}}{t_{MPS}}=\frac{1}{m-1} \end{array}$$

**Figure 11:**
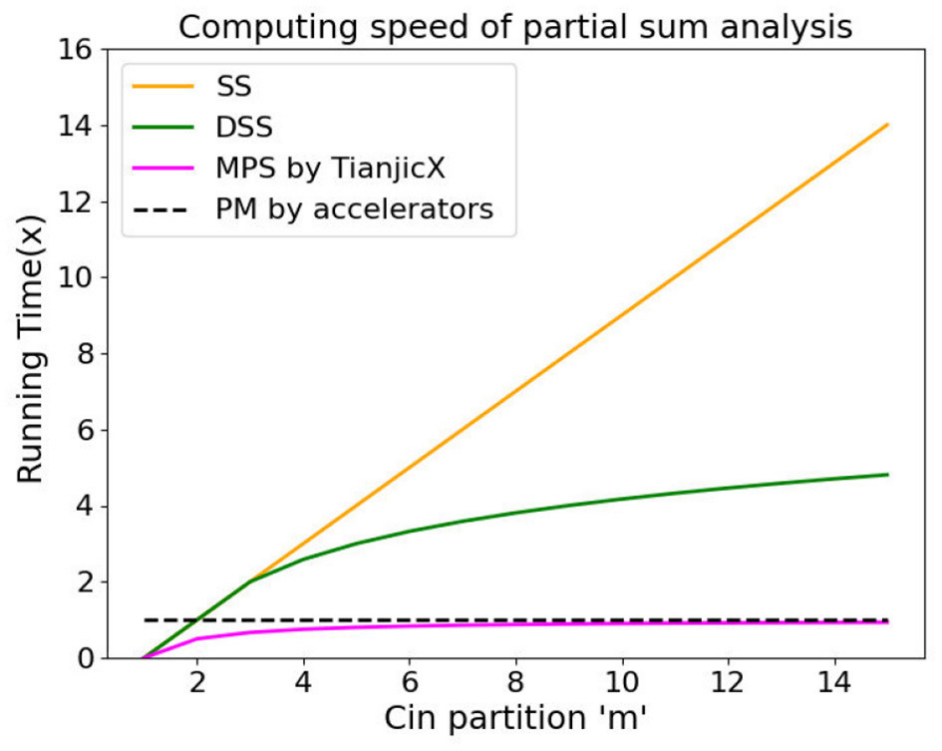
The running time of the methods for processing the Psums.

Here, *t*_*SS*_, *t*_*DSS*_, *t*_*PM*_, and *t*_*MPS*_ represent the execution times under the SS, DSS, PM, and MPS methods, respectively. The simulation results are presented in Figure 11. The SS method is employed by the mixed-signal in-memory computing chip proposed in Le Gallo et al. (2023). The DSS method, based on the Wallace tree multiplier, has been widely adopted by many designers (Lakshmi et al., 2021; Solanki et al., 2021; Srinivas and Umapathi, 2022). The PM method, on the other hand, is commonly utilized by accelerators (Shao et al., 2019; Chen et al., 2019; Jouppi et al., 2017; Sze et al., 2017; Yin et al., 2017; Kung et al., 2019).

From the simulation results, it is evident that the MPS method achieves the shortest runtime compared to the other approaches. In particular, the MPS method, implemented by TianjicX platform, demonstrates significant superiority in enhancing the computation speed of Psums compared to the PM method, which is widely adopted by many accelerators. Specifically, the number of processing elements (PEs) in the accelerators is 16, corresponding to a partitioning of *C*_*in*_ into 16 groups. MPS demonstrates enhanced runtime performance when utilizing 16 PEs, achieving a reduction in computational latency of 6.7% compared to the PM, as delineated in Equation 13. Furthermore, the speedup factor displays an inverse relationship with the number of PEs, thereby attaining the highest efficiency gains at this configuration.

### 5.3 Experiment of spatial-temporal density mapping

The layer-wise mapping approach is widely utilized by accelerators and neuromorphic chips in a pipelined manner (Zimmer et al., 2020; Pei et al., 2019; Le Gallo et al., 2023). Consequently, we use layer-wise mapping as the baseline for comparison. To evaluate the performance of the proposed fine spatial-temporal density mapping scheme, we designed multiple experimental groups with varying spatial-temporal density variances while keeping the total number of allocated cores constant. The allocation of cores and the coupling layer configurations for these groups, corresponding to the two types of networks, are summarized in Tables 2, 3.

**Table 2:** Different coupling mapping schemes of designed networks.

**# cores**	**Layer-wise**	**2-layers-c**	**3-layers-c**	**6-layers-c**
Layer 1	7			
Layer 2	14	24		
Layer 3	7		28	
Layer 4	7	8		56
Layer 5	14			
Layer 6	7	24	28	
Aggregation	56	56	56	56
σ_ρ_	629.4	69.8	0	0

**Table 3:** Different coupling mapping schemes of ResNet-50 (2b,2c).

**# cores**	**Layer-wise**	**2-layers-c**	**3-layers-c**	**6-layers-c**
Layer 1	14			
Layer 2	28	42		
Layer 3	14		56	
Layer 4	14	28		112
Layer 5	28			
Layer 6	14	42	56	
Aggregation	112	112	112	112
σ_ρ_	184.1	43	0	0

In Tables 2, 3, the abbreviation x-layers-c denotes the use of *X* coupling layers. By applying the multi-layer coupling method, the spatial-temporal density and the variance σ_ρ_ of core utilization can be adjusted. For instance, as shown in Table 2, under the layer-wise mapping scheme, the computational tasks for each layer are distributed across 7 cores, 14 cores, and 7 cores, respectively. By systematically varying the spatial-temporal density, we conducted experiments using different mapping schemes, including a 2-layer mapping scheme, a 3-layer mapping scheme, and a 6-layer mapping scheme.

**Figure 12:**
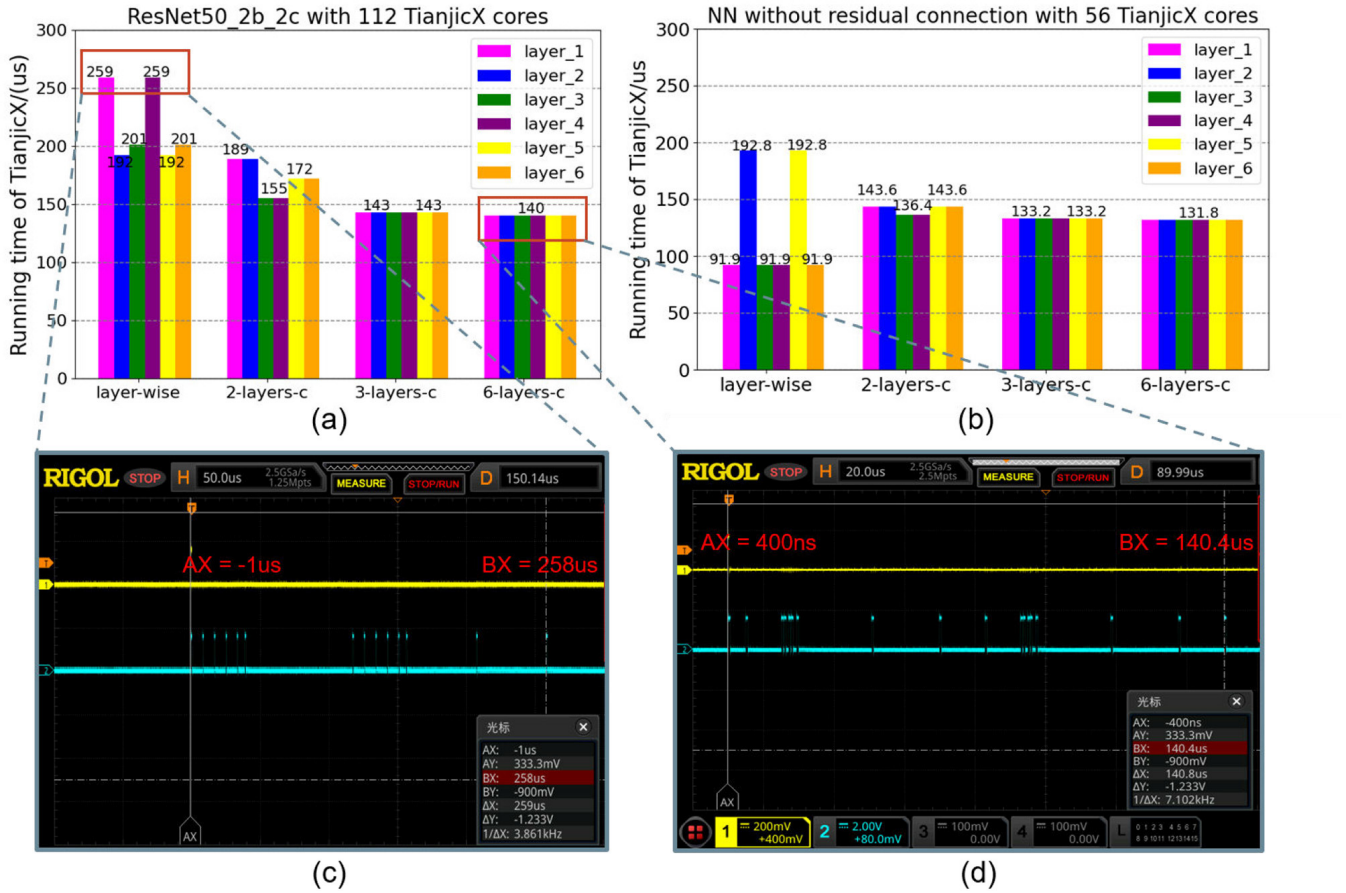
The running time and latency of different spatial-temporal densities mapping: **(a)** two blocks of ResNet-50; **(b)** two blocks without residual connection. **(c)** the runtime of chip under the layer-wise mapping tested by the oscilloscope. **(d)** the runtime of chip under the multi-layers-coupling mapping tested by the oscilloscope.

The results, presented in Figure 12, demonstrate that reducing the spatial-temporal density variance σ_ρ_ leads to decreases in both execution time and tail latency. In the layer-wise mapping scheme, the computation times for the first and third layers were measured at 259μ*s* using an oscilloscope. Similarly, the computation times for the second and fifth layers were recorded at 192μ*s*. Notably, the total computation time for the sixth layer, from initiation to completion, was also 259μ*s*. In contrast, under the 6-layer coupling scheme illustrated in Figure 12a, where all six layers are interconnected, the computation time for each individual layer was consistently reduced to 140μ*s*. All runtime data for these chips were acquired through oscilloscope measurements. Figures 12c, d present two examples, and similar data can be obtained from the provided materials. As a result, the computation speed under the 6-layer coupling scheme was improved by a factor of 1.85 compared to the layer-wise mapping scheme. Furthermore, the tail latency was completely eliminated in the 3-layer coupling and 6-layer coupling mapping schemes.

This improvement is attributed to the fact that no reshaping is required between adjacent layers, as the dimensions of partitions within each layer remain consistent under the 6-layer coupling mapping scheme. Additionally, the output activations in the coupling layers are stored in local memory, eliminating the need for communication between layers. This significantly reduces the proportion of time dedicated to data communication during task execution, thereby enhancing the computational efficiency of the hardware. Since large-scale networks can be divided into multiple flow blocks, this density mapping technique can be broadly applied to full-scale implementations of ResNet-50 and other larger neural networks.

However, it should be noted that full layer-coupling mapping is not universally optimal. Although coupling all layers can effectively eliminate the tail latency of each core by reducing σ_ρ_, it may have adverse effects on computation time and overall latency. As the number of coupled layers increases, the partitioning scheme becomes more complex and less efficient. This leads to increased reshaping latency and a significant reduction in MAC utilization efficiency. Therefore, a trade-off exists between the number of coupled layers and the value of σ_ρ_ in spatial-temporal density mapping. This trade-off is ultimately determined by the specific hardware design.

## 6 Conclusion

In this work, we propose spatial-temporal density mapping for the first time that leverages computational resources and spatial resources of a many-core chip. Furthermore, we propose the Negative Sequence Memory Management (NSM) approach to improve space utilization. The NSM can improve space utilization by average 3.05 times compared with the (PSM) used by many-core systems. And we propose the Many-core Parallel Synchronous (MPS) approach to improve the computational speed. It is demonstrated that the MPS can be improved by 6.7% compared to the Pipelined Method (PM) which is adopted by the many-core systems. To demonstrate the superior performance of Spatial-Temporal density Mapping with these optimized approaches, we implement the mapping methods on our state-of-the-art many-core chip, TianjicX. Intensive experiments show that using fine spatial-temporal density mapping improves performance by 1.85x compared to layer-wise mapping used by many-core systems. We believe that optimizing methods for fine spatial-temporal density mapping can help establish a general and efficient mapping framework for many-core systems with variable spatial-temporal density.

## Data Availability

The raw data supporting the conclusions of this article will be made available by the authors, without undue reservation.
